# Software Defined Radio (SDR) and Direct Digital Synthesizer (DDS) for NMR/MRI Instruments at Low-Field

**DOI:** 10.3390/s131216245

**Published:** 2013-11-27

**Authors:** Aktham Asfour, Kosai Raoof, Jean-Paul Yonnet

**Affiliations:** 1 Grenoble Electrical Engineering Lab (G2E-Lab), BP 46, Saint Martin d'Hères 38402, France; E-Mail: Jean-Paul.Yonnet@g2elab.grenoble-inp.fr; 2 Laboratoire d'Acoustique de l'Université du Maine, Rue Aristote, Le Mans Cedex 09 72085, France; E-Mail: kosai.raoof@univ-lemans.fr

**Keywords:** SDR, DDS, NMR, MRI, low-field

## Abstract

A proof-of-concept of the use of a fully digital radiofrequency (RF) electronics for the design of dedicated Nuclear Magnetic Resonance (NMR) systems at low-field (0.1 T) is presented. This digital electronics is based on the use of three key elements: a Direct Digital Synthesizer (DDS) for pulse generation, a Software Defined Radio (SDR) for a digital receiving of NMR signals and a Digital Signal Processor (DSP) for system control and for the generation of the gradient signals (pulse programmer). The SDR includes a direct analog-to-digital conversion and a Digital Down Conversion (digital quadrature demodulation, decimation filtering, processing gain…). The various aspects of the concept and of the realization are addressed with some details. These include both hardware design and software considerations. One of the underlying ideas is to enable such NMR systems to “enjoy” from existing advanced technology that have been realized in other research areas, especially in telecommunication domain. Another goal is to make these systems easy to build and replicate so as to help research groups in realizing dedicated NMR desktops for a large palette of new applications. We also would like to give readers an idea of the current trends in this field. The performances of the developed electronics are discussed throughout the paper. First FID (Free Induction Decay) signals are also presented. Some development perspectives of our work in the area of low-field NMR/MRI will be finally addressed.

## 1. Introduction

The development of Nuclear Magnetic Resonance (NMR) and Magnetic Resonance Imaging (MRI) systems is still motivated by the outstanding features of this technique. Medical diagnosis and medical research as well as analytical chemical spectroscopy are the main areas that have mostly benefited from the potentials of the NMR. In these areas, high magnetic fields (up to 11 T), generally produced by superconducting magnets, are frequently used. This race to high fields is obviously justified by the high signal-to-noise ratio and the high resolution of the image and of the spectra that could be achieved.

Low-field (about 0.1 T) NMR is also well-known now. When compared to high field, low-field NMR has the main advantage of reduced system cost, size and complexity since low fields can be easily generated by electromagnets or permanent magnets. These features have allowed the technique to conquer exciting new applications, especially for situations where the bulky and prohibitively expensive high field systems cannot be used or the sample to be studied cannot be moved. The applications of low-field and mobile NMR systems could include—but are not limited to—areas such as material science, non-destructive quality control of products (food, polymers…), moisture and porous media measurement, paper, wood and oil industries, non-destructive studies of cultural heritage (stones, old master painting, mummies…). Even biomedical applications (MRI/NMR of the tendon or skin) should benefit from mobile and low-field instruments. An excellent review of many of these promising applications of the NMR has recently been presented in [1].

Low-field NMR spectrometers are, in general, not commercially very renowned, despite the existence of some worthy and valuable commercial systems such as those from Magritek, Inc. (Aachen, Germany) for example. Nevertheless, the demand for dedicated spectrometers is still increasing. One of the difficulties associated with such systems is that it may be often necessary to perform a new hardware and software design and development to optimize the whole system for each specific application. The design of the hardware (transmitter and receiver) is crucial and it requires cautions. However, while many publications (academic courses, books, articles…) deal with the design of the radiofrequency (RF) coil itself, which obviously defines the final signal-to-noise ratio, these publications rarely address issues for the design of the transmitting and receiving electronics. This electronics is usually assumed to be a “black box”. Some groups have already worked to develop their own dedicated low- and very-low field NMR spectrometers for specific applications [2–8]. For example, we have recently proposed very low-field NMR spectrometers that allow detection of the ^1^H NMR signals at 4.5 mT (about 180 kHz of Larmor frequency) without pre-polarization [2–4]. Such developments were initially motivated by their application to the measurement of the *absolute polarization* of hyperpolarized xenon (^129^Xe). These spectrometers were based on the use of general-purpose commercial data acquisition boards (DAQ) from National Instruments (Austin, TX, USA). Based on DAQ boards, an NMR system at 0.1 T for educational purposes was also presented in [5]. Another NMR spectrometer with fully analog electronics has been proposed for the NMR of hyperpolarized helium (^3^He). Other works carried out in [7,8] were focused on the realization of a digital home-built NMR spectrometer working at high field (frequencies up to 80 MHz). In these designs, the NMR system was composed of a form of combination of analog and digital circuits. Actually, analog mixers were necessary in the transmitter and/or before digitalization by the receiver. Despite the great merits and the advances achieved by these works, large spread of the low-field NMR still requires decreasing the complexity of the hardware and associated software, increasing the flexibility as well as lowering the cost of the system. A main idea is that hardware and software of the spectrometer could be easily updated for the largest number of applications and different working frequencies with minimum development time and low cost.

In our laboratory, we are currently working on the design of low-field and mobile NMR instruments for industrial applications such as the quality control of oranges, the moisture measurement in nuts or other products as well as the inside-out NMR for high resolution spectroscopy in inhomogeneous magnetic fields [9]. Our work includes the design of the full system, *i.e.*, the magnet and the spectrometer [10].

The present paper will mainly focus on a new design of the electronics for transmitting and signal receiving. It actually reports some parts of our experience in this area. This experience is presented here through the development of a fully digital RF hardware and the associated software for the NMR at 0.1 T (4.2 MHz of Larmor frequency). This electronics is based on three key elements: a Direct Digital Synthesizer (DDS) for pulse generation, a Software Defined Radio (SDR) for signal receiving and a Digital Signal Processor (DSP) as a controller and pulse programmer. Except for the RF power amplifier and the low-noise preamplifier, the proposed system is fully digital and programmable by software. Its architecture is open so as to allow for the easy update and extension for a large number of NMR experiments and working frequencies. One of the ideas of this paper is to enable the NMR systems to “enjoy” from existing advanced technologies that have been achieved in others research areas. Actually, the main concepts of this new developed system are inspired from advanced digital signal processing techniques that are now frequently employed in telecommunication systems, especially in mobile radio and cellular base stations. The paper attempts to show that these techniques could be applied to the NMR through the realization of a proof-of-concept.

We should however note that some devices which may have similar architectures have been available from specialized manufacturers for a few years now. For example, the Redstone™ spectrometer from Tecmag (Houston, TX, USA) has a modular design with up to 128 transmitters and 512 receivers [11]. Imaging Technology Abruzzo S.r.l (L'Aquila, Italy) proposes another console with four receiving channels [12]. Nevertheless, one goal of the paper is actually to help people to get an idea of the current trends in this area. Another goal of this paper is to try to make the NMR systems versatile and easy to replicate so as to help developers and research groups in realizing NMR spectrometers with flexibility, low cost and minimum development time. This is why we describe with some details the various aspects of the realization. While some of these aspects may seem basic for knowledgeable developers, we believe that they may be useful for those who would like to learn about the conditioning electronics and the application of telecommunication technology to NMR sensors.

In addition to the presentation of the new developed system (DSP, DDS and SDR) and the associated software, the expected performances and potentials of the system are given throughout the paper. First experimental tests of the receiver are then presented. Some issues related to the design of the RF coil and the adequate tuning and matching circuits at low-field are illustrated, together with the first Free Induction Decay (FID) for validation purposes. Development perspectives of our work in area of sensors for the low-field NMR will also be addressed.

## 2. Hardware Implementation

**Figure 1.**
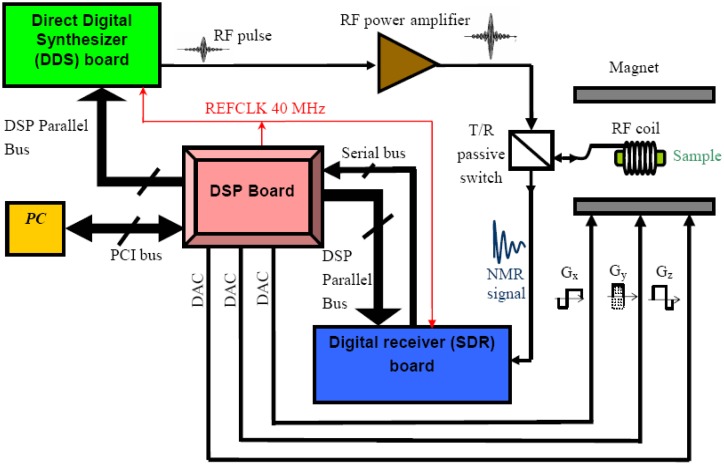
A block diagram of the DDS and SDR based NMR system at 0.1 T.

Figure 1 shows the hardware of the home-built system. The core of the design is a Digital Signal Processor (DSP) board which controls the other two elements of the electronics: the Direct Digital Synthesizer (DDS) and the Software Defined Radio (SDR) or digital receiver. These elements were designed as daughter boards of the DSP and are fully programmable by the DSP via its parallel external bus.

The excitation pulse is synthesized by the DDS. After amplification by a home-built RF power amplifier (about 250 W peak), this pulse is sent to the well-tuned RF coil via a transmit/receive (T/R) passive switch. At the end of the excitation pulse, the NMR signal is detected by the coil (assuming a transmit-receive coil) and transmitted, via the same T/R switch, to the digital receiver. This receiver includes an amplifier and a Software Defined Radio (SDR) for digital quadrature demodulation, decimation and filtering. The samples of the demodulated signals are then transmitted to the DSP, via a serial bus, where more signal processing could be performed if necessary.

For imaging purposes, the gradient signals are generated by the DSP board thanks to a set of three Digital-to-Analog Converters (DAC). Furthermore, the DSP, DDS and the SDR are synchronized with a same external 40 MHz reference clock (REF CLK) available on the DSP board. The key elements of this design and their main performances are addressed in the next sections.

### 2.1. The Digital Signal Processor (DSP) Board

The DSP was designed as PCI plug and play board. This home-built board includes mainly the processor ADSP-2106x SHARC—Super Harvard Architecture Computer—from Analog Devices (Norwood, MA, USA) [13]. The reputation of the high performance ADSP2106x processors comes mainly from their ability to perform 32-bits floating-point calculations. They are optimized for real-time digital signal processing and are widely used in applications requiring high computing speed. Compared to a conventional processor, the use of DSPs in the MRI system offers more options and flexibility in signal processing and image reconstruction in real time as well as in the time management during the sequence.

The processor has an on-chip internal memory of 4 Mbits for the temporary storage of the NMR signal samples. It has also several fast and efficient protocols for communication and data transfer with a wide variety of devices (external memory, PC, other DSP…). In particular, external parallel buses of the processor were used in our design to control and to upload the parameters of the SDR and the DDS. These chips were actually memory- mapped devices of the DSP. The high speed synchronous serial port of the DSP was used to transfer the digital data (NMR signal samples) from the digital receiver to the DSP. External channels are available to transfer data from the DSP memory to the host (PC, other DSP…).

In addition to the processor, the DSP board includes also the set of three 16-bit DACs. The level of the analog signals at the output of these DACs could be adjusted in the range ±10 V. The residual noise output is less than 1 mV. These DACs are used for the generation of the gradient waveforms at a sampling frequency up to 2 MHz.

The DSP, which ensures the role of the pulse sequencer, is programmed in assembly language as it will be presented is Section 3. In our design, the processor is clocked at 40 MHz. This 40 MHz clock (REFCLK) is also used as a reference clock for the DDS and the SDR (Figure 1).

### 2.2. The Direct Digital Synthesizer (DDS)

The transmitter board is home-built. Its core is the DDS AD9852 from Analog Devices [14]. The DDS technology is based on the fact that one could define a sinusoid with known frequency, amplitude and phase by specifying a set of values (samples) taken at equal intervals defined by the sampling frequency. This series of values is supplied to a Digital-to-Analog Converter (DAC) that provides the analog signal. This technology is now widely used for in telecommunication systems.

**Figure 2.**
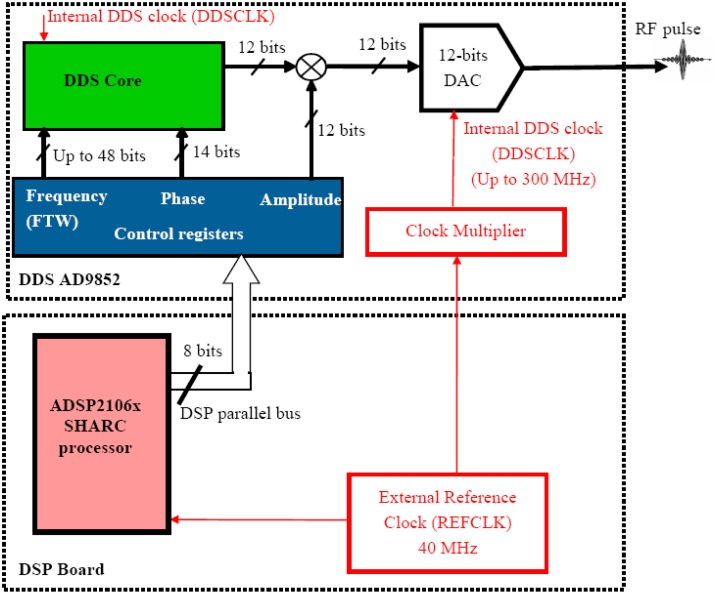
A simplified diagram of the DDS and its interface with the DSP.

Figure 2 shows a simplified diagram of the DDS AD9852 and its interface with the DSP. When referenced to an accurate clock source, the DDS, coupled with an internal high speed DAC, generates a highly stable and accurate frequency-phase-amplitude- cosine waveform. The circuit allows theoretically the generation of output signals at frequencies up to 150 MHz when it is clocked at 300 MHz.

In our design, we make use of the clock multiplier to generate an internal DDS clock (DDSCLK) from a lower frequency external reference clock (40 MHz). In addition to the fact that the whole system should be synchronized with a same clock, the use of the clock multiplier allows the developer to avoid difficulties of implementing a high frequency clock source system. We used a multiplication factor of 4 so as the final frequency of the internal clock of the DDS is 160 MHz. This frequency is also the sampling frequency of the on-chip 12-bits DAC. The value we have chosen for this frequency was actually a trade-off between the generation of cosine waveform with high resolution and spectral purity and the overheating of the chip.

The frequency, phase and amplitude are digitally programmable by writing values in the DDS control registers through the 8-bit parallel external bus of the DSP. The desired output frequency, F, is translated to a Frequency Tuning Word (FTW) according to Equation (1):

(1) $$\text{FTW}=2^{N}\frac{F}{\text{DDSCLK}}$$

where N is the frequency resolution (up to 48 bits), FTW is a decimal number, DDSCLK is the frequency of the DDS clock and F expressed in Hertz.

High frequency resolution can be obtained. For example, if DDSCLK = 160 MHz and N = 34 bits, the obtained frequency resolution is about 0.009 Hz. In our implementation, this 34-bit resolution was chosen for synchronization purposes and exact match between the frequency of DDS and the demodulation frequency (frequency of the NCO) of digital receiver (see Section 2.3).

In a similar way, the output phase and amplitude can be defined digitally with resolutions up to 12-bit and 14-bit, respectively. Shaped (amplitude modulated) RF pulses are easily generated by making use of a 12-bit digital multiplier which allows user to dynamically and digitally modulate the amplitude of the output signals. This allows generation of almost arbitrary RF pulse shape. Frequency-and phase-modulated signals can also be easily generated.

This DDS is actually a multifunction and a highly flexible device that addresses a wide range of important and required functionalities for the NMR systems. In addition to the high resolutions that could be achieved and the classical amplitude modulation, the DDS can easily satisfy more sophisticated and new emerging forms of NMR excitation such as shim-pulses (extension of the concept of uniform broadband excitation). These pulses that require phase- and amplitude- modulation of the RF pulse together with a modulation of the gradient tensor are promising excitation forms for the high resolution NMR in inhomogeneous magnetic fields [1]. Furthermore, most of the NMR techniques need that RF source of the transmitter has the agility to rapidly switch its phase, frequency and amplitude. For the used DDS, the update rate of these parameters is user programmable and could be as fast as 6 ns.

### 2.3. The Software Defined Radio (SDR) for Signal Receiving

Another key element of the NMR/MRI system we built is the digital receiver. As we have mentioned in the Introduction, the architecture of the receiver is inspired from advanced digital signal processing techniques that are especially applied in mobile radio and cellular base stations. In the area of telecommunication, the set of these techniques is commonly called *Software Defined Radio (SDR)* or *digital receiving*. Its basic idea is to perform the digital-to-analog conversion as close to the receiving coil as possible. All the signal processing (quadrature demodulation, decimation, filtering) can then be realized in the digital domain. This has the advantage of noise and distortion reduction associated with analog mixing stage and better out-of-band noise rejection by the decimation and the digital filters. Moreover, the parameters of the system are fully programmable. They could be modified by the software and without changing the hardware architecture.

The receiver we built was inspired from an evaluation board of the dual Digital Down Converter Chip CLC5902 from National Semiconductor (Santa Clara, CA, USA) [15]. This evaluation board was factory designed to be programmable by the PC through the serial RS232 port, an on-board microcontroller and associated software for applications in cellular base stations, satellite receivers and digital communication.

**Figure 3.**
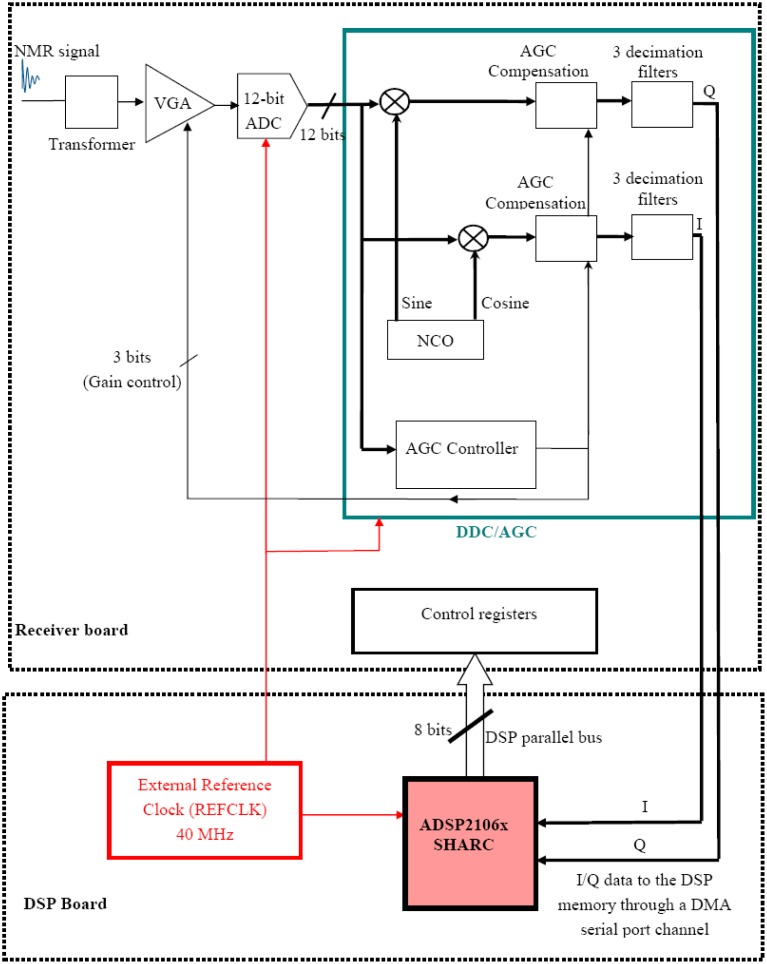
Block diagram of one receiving channel and its interface with the DSP. The second receiving channel is not shown.

We have re-designed this evaluation board to integrate it in our home-built NMR system (together with the DDS and the DSP boards). Hardware modifications and new software development were necessary. These modifications have allowed programming the receiver directly by the parallel port of the DSP with suitable new software and data transmission through the high speed serial port of the DSP as well as synchronization of the whole system with a same reference clock (40 MHz) for the NMR experiments. Figure 3 shows the block diagram of the architecture of one receiving channel with the DSP. The second receiving channel is identical (not shown in Figure 3) and is not currently used in our applications. Extension to more than two channels could be possible.

The receiver channel mainly consists of a Variable Gain amplifier (VGA), an Analog-to-Digital Converter (ADC) and Digital Down Converter (DDC) with an Automatic Gain Controller (AGC). It accepts intermediate frequency (IF) analog signals (NMR signals in our case) and performs a digital quadrature demodulation to obtain the final In-phase (I) and Quadrature (Q) signals.

The channel input is coupled into the VGA by a transformer. This transformer matches the input impedance of the VGA (200 Ω) to the 50 Ω input connector and the NMR coil. The signal is then amplified by the low-noise and wideband VGA. This VGA has a digitally controlled gain range from −12 dB to +30 dB. It is also the gain control element of an Automatic Gain Control (AGC) loop.

After amplification, the signal is directly sampled by a 12-bit Analog-to-Digital Converter (ADC) clocked at 40 MHz. It is a 12-bit and wideband ADC capable of inputs as high as 300 MHz at sample rates up to 70 MHz. The sampled signal is applied to the Digital Down Converter (DDC) which integrates the AGC controller. The DDC uses a Numerically Controlled Oscillator (NCO) to generate the sine and cosine digital waveforms used by the digital mixer. The frequency and phase of the NCO are set by loading the appropriate control registers. This loading is done by the DSP. In a similar way as for DDS (Equation 1), the Frequency Tuning Word for the NCO (FTW_NCO_) is 32-bit and is given by Equation (2):

(2) $${\text{FTW}}_{\text{NCO}}=2^{32}\frac{\text{F}}{\text{REFCLK}}$$

where FTW_NCO_ is a decimal number, REFCLK is the frequency reference clock and F is the frequency expressed in Hertz. For REFCLK = 40 MHz, the obtained resolution is about 0.009 Hz.

In NMR, it is necessary to exactly match the frequencies of the NCO and the DDS. The same frequency resolution, expressed in Hertz, has to be obtained. As the NCO and DDS are clocked at different rates, it was necessary to use a 34-bit Frequency Tuning Word for the DDS. In the same way, the phases of the NCO and DDS must have the same resolution. This was straightforward to implement in our system by software. Furthermore, thanks to programming features of both NCO and DDS and the use of a unique reference clock for the whole system, the receiver remains phase locked with the transmitter. This is mandatory in NMR sequences, especially to make signal averaging possible.

The digital mixers of both channels of the DDC are followed by digital AGC compensation which is controlled by the AGC controller. This controller measures the output level of the ADC (output power measurement using an envelope detector). This measurement is used to automatically adjust the VGA gain so as the dynamic range of the input signal is expanded or compressed to fit the full scale of the ADC. By doing so, the dynamic range of the ADC is extended. This feature is particularly useful for the MRI where the signals (echoes for example) have generally very different levels. The AGC ensures optimization of the gain and the ADC dynamic range for each signal (despite the fact that the ADC may never see full scale in the case of the weak NMR signals). After digitization, it is however necessary to compensate for the compressed or expanded dynamic range. This compensation is digitally performed by the AGC loop (Figure 3). It is also possible to inhibit the AGC loop and force fixed VGA gain values. More details about the AGC operation could be found in [15].

After the digital AGC compensation, the I/Q signals are filtered by three decimation filters that achieve a low-pass filtering and low output sample rate. The first filter is a Cascaded-Integrator-Comb (CIC) with fixed coefficients. It decimates (under-samples) the input data by a programmable factor between 8 and 2,048. The CIC filters are widely used in digital receivers. Their structure is very simple and their implementation requires no multipliers and only limited storage. Consequently, the computation speed is very fast. These characteristics make them especially suitable as the first-level decimator filters for high sample rate change. The CIC output is followed by two filtering stages. The first stage is a 21-ceofficients symmetric Finite Impulse Response (FIR) filter with programmable coefficients. It also decimates the input by 2 (fixed decimation factor). The second filter is also a symmetric FIR with user programmable coefficients (63 coefficients). It decimates by a factor of 2 or 4.

The operating frequency of our NMR system is 4.2 MHz (0.1 T of magnetic field). This signal will actually be over-sampled (at 40 MHz) by the ADC. An advantage of this high over-sampling rate is the potential for processing gain. Processing gain usually refers to the increase of the ADC Signal-to-Noise Ratio (SNR) and dynamic range that can be obtained by the decimation. Generally, the ADC SNR is limited by the thermal noise. For a given ADC, the noise bandwidth is normally defined as the Nyquist bandwidth (the half of the sampling frequency). For the ADC we used and for a sampling frequency of 40 MHz, this yields to an SNR of at least −65 dB relative to the full-scale (dBFS) [16]. The decimation technique that reduces the sampling frequency of the I/Q base band signal provides a much narrower final bandwidth at the output. Since the noise is spread out over the full bandwidth of the digitizer, only the noise in narrower bandwidth is retained. For example, if the final output bandwidth is 25 kHz (±12.5 kHz) and the initial sampling frequency is 40 MHz, one can expect an ADC SNR improvement given by Equation (3):

(3) $$\text{Processing gain}=10\times \text{log}\frac{25\text{kH}}{25\text{MkH}}=-29\text{dB}$$

The expected ADC output SNR (after the decimation filters) is then given by Equation (4)

(4) SNR(dBFS)=−65dBFS−29dB=−94dBFS

We have to mention that this SNR will not be realized in most NMR experiments. Actually, the Equation (3) does not take into account the thermal noise from the receiving coil and the preamplifier which could be more important than the other noise sources (ADC and quantization noises). The level of this thermal noise could be of several least-significant bits. One could however expect some improvement in the SNR because the well designed digital filters will be very effective in out-of-band thermal noise rejection.

## 3. Sequencer and Software

**Figure 4.**
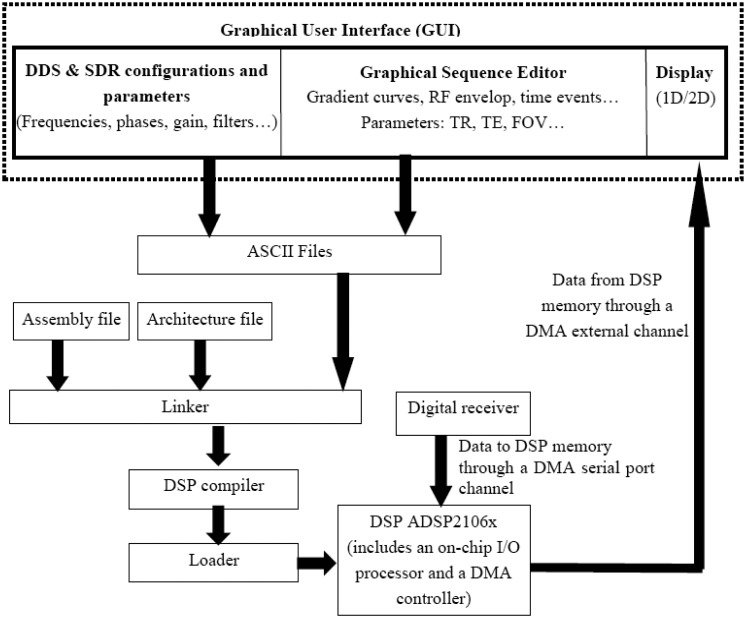
Block diagram of the developed software.

While the present paper is only focused on a proof-of-concept of the use of digital concepts, the software was thought to already take into account the future implementation of almost all types of NMR/MRI sequences. This software was developed using the LabWindows/CVI^©^ environment and DSP assembly language [13]. Figure 4 illustrates its block diagram. The Graphical User Interface (GUI) allows user to define and edit all the configurations and parameters (frequencies, phases, filters…) of the hardware (DDS and SDR). It also enables user to draw and/or graphically edit a sequence (pulse envelop, gradient waveforms) and to define parameters such as repetition time (TR), echo time (TE) and the Field-of-View, *etc*. The use of this graphical sequence editor allows avoiding programming the gradient waveforms in text sequence editor.

Data from the GUI are stored in ASCII files. These files are then linked with the assembly and the architecture files of the DSP. The assembly compiler provides then an executable file which is loaded in the DSP RAM via the PCI bus. This assembly program or sequence program interprets all the data defined in the GUI and controls all the temporal events of the sequence and the data transfer and commands between the three boards (DSP, DDS and SDR). The use of the assembly language was adequate to optimize the memory use of the DSP and to facilitate the management of the temporal events within the sequence. In this way, the DSP ensures the role of a high performance pulse programmer (sequencer) which is able to execute each program instruction at every clock cycle with a high temporal resolution (25 ns for a clock of 40 MHz). The generation of gradient signal is achieved with 0.5 μs of temporal resolution.

It is important to note that a lot of the sequence and DDS/SDR parameters could be modified in real time during the sequence execution without reloading the program in the DSP. This important functionality was naturally implemented thanks to the input/output (I/O) registers of the DSP. The host of the DSP (the PC in our case) can directly write and/or read theses registers which are continuously read by the DSP at certain interrupt services.

The management of the data transfer from the digital receiver to the DSP and from the DSP to the host PC requires some cautions. This management has been achieved relatively easily thanks to the high performances and high flexibility of the ADSP210x DSP. Actually, the architecture of this chip is composed of four main parts: a Core Processor, a Dual-Ported SRAM, an External Port and an Input/output (I/O) Processor [13]. This on-chip I/O processor is responsible for managing a Direct Access Memory (DMA) Controller. This DMA controller handles several transfer channels. Two of these channels belong to the synchronous serial ports and are used to transfer the received data from the digital receiver to the memory of the DSP (Figures 3 and 4). Once it has been setup, the DMA controller operates independently from the core processor [13]. This frees the core processor to continue with other tasks (like the generation of a next RF pulse if required in the sequence).

Acquired data could be available to the host PC immediately when a block of the data (with user-defined size) is available in the DSP memory. The used DSP has a very powerful host interface which is interfaced to the PCI bus of the PC by using a PCI target I/O accelerator bridge on the DSP board. The host can read the data as soon as they are received and at each moment independently from the core processor. This transfer between the DSP memory and the host is done, once again, by the DMA controller of the I/O processor. Actually, in addition to the serial port channels, the DMA controller manages external channels which are responsible for data transfer between the DSP memory and the external hosts via the powerful host interface.

Since the data transfer from the DSP to the host is managed by the DMA controller of the I/O processor (and not by the core processor), it could be done when the data (with user-defined size) are available. It can also be done at the end of the sequence or during its dead time. In our design, managing the moment at which one wants the data to be available to the host is a task of the sequence developer. This moment may be defined with some flexibility according to the constraints of each sequence.

The whole structure of the software is actually very flexible and open so as to allow an ease-of-use of existing sequences and low-complexity implementation of new sequences. Other required functionalities for NMR system were also implemented in the same GUI (not shown in Figure 4). These include functions for coil tuning and for active shimming.

## 4. Discussions and Validation

### 4.1. First Performances and Test of Receiver

As it was mentioned, one of the important advantages of the SDR is its completely programmable feature. This allows user to easily choose parameters such as amplifier gain, decimation factors and filters coefficients to obtain optimum performances for a given experience.

In order to test the receiver operation, we fixed the working frequency at 4.2 MHz (Larmor frequency at 0.1 T). The sample rate of the ADC was 40 MHz. The CIC decimation factor was fixed at 400. The sample rate at the output of the filter was then of 100 kHz.

**Figure 5.**
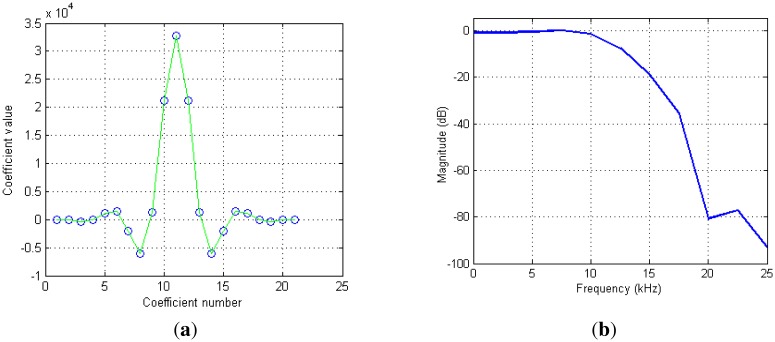
(**a**) The impulse response of the first programmable filter. (**b**) The frequency response of the same filter. The filter has a fixed decimation factor of 2. In this example, the input and the output sample rates were 100 kHz and 50 kHz, respectively.

The two programmable FIR filters were designed. This first one was mainly designed to compensate for a slight droop induced by the CIC filter. It improves so the flatness of the CIC pass band. In addition, it could provide stop band assistance to the following filtering stage. Figure 5 shows an example of the impulse and frequency responses of a set of coefficients we used in our system.

The filter has a unit gain and flat pass band (only 0.01 dB ripple) over nearly the full output band pass with 70 dB of out-of-band rejection (Figure 5b). Notice also that impulse response is symmetric. Only 11 coefficients (over 21) are actually loaded in the receiver.

**Figure 6.**
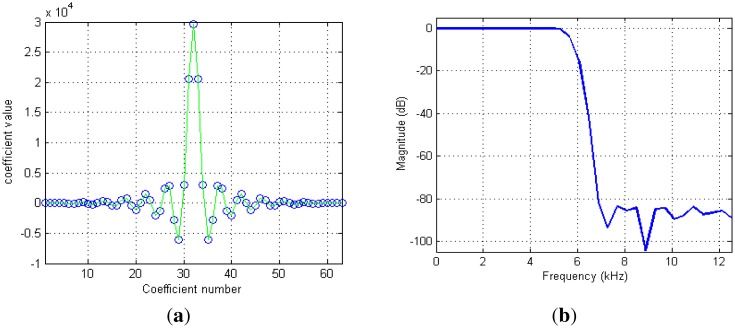
(**a**) The impulse response of the second programmable filter. (**b**) The frequency response of the same filter. The filter has a programmable decimation factor (2 or 4). In this example, the input and sample rate was 50 kHz. The decimation factor was programmed to 2. The output sample rate was 25 kHz.

The second FIR filter is responsible for limiting the final bandwidth. It ensures also a high out-of-band noise rejection. An example of the impulse and frequency responses of this filter is shown in Figure 6. The filter has 0.03 dB of pass band ripple and about 80 dB of out-of-band rejection. The final cut-off frequency is defined mainly by this filter. In our case, it was fixed to about 5.8 kHz at −3 dB. This frequency corresponds simply to the bandwidth we have fixed for NMR signals during future image acquisition with basic spin- and gradient-echo sequences in our system. This bandwidth is naturally related to the maximum image resolution.

It is very important to note that digital decimation filters introduce actually a temporal group delay which depends on the filters order, the overall decimation factors and the system clock period.

This delay must still be present in the mind of the sequence developer and have to be taken into account, especially in situations where RF pulses and acquisition windows are very close each. Actually, in these situations, the DSP must be able to manage at the same the generation of the RF pulse and the receiving of the delayed data. In our design, this problem was solved thanks the on-chip I/O processor and the independent DMA controller offered by the ADSP2106x as it was mentioned in Section 3. Moreover, it is also very important to mention here that the filtering and decimation are done inside the digital receiver and not by the DSP. This help a lot to have an adequate distribution of the calculation power between the calculation units.

We note finally that if simultaneous RF transmission and delayed data receiving should occur, the analog noise from the transmitting section will not affect the data which are already digital. Actually, a main advantage of the direct sampling and the digital receiving is that analog noise has no more effect after the analog-to-digital converter (ADC), unless the RF, travelling by air or on the ground, is so high so as to invert digital bits. In all the cases, the layout of the whole system and grounds should be designed with cautions. In our design, separate analog and digital grounds are used for each of the DSP, DDS and SDR boards.

Under these conditions, experimental tests have been conducted to verify some of the performances of the receiver. The VGA gain was fixed to 30 dB (the AGC loop was inhibited). First, a pure sine wave of 4.2 MHz is applied with variable amplitude from –50 dB to –130 dB to the receiver input. The observed sensitivity (the smallest observable signal) for the receiver was –120 dB and the best dynamic range for the digital full scale output was at –96 dB. Noise rejection was also evaluated by introducing a sum of 4.2 MHz sine wave and a large band noise with a zero signal-to-noise ratio. The results have shown a total rejection out-of-band noise which was beyond our measurement instrument.

We note finally that NMR spectrometer could also be used at working frequencies as high as 150 MHz (for both DDS and SDR), without any change of the hardware (except for the tuned coil) and without the use of any analog mixer as it was the case in [7].

### 4.2. The Complete NMR System and First NMR Signals

**Figure 7.**
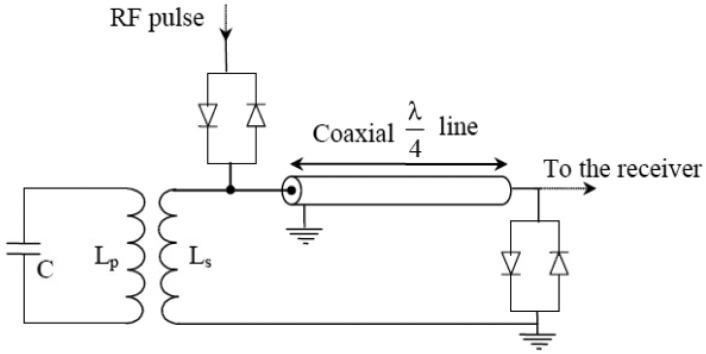
The electrical circuit of the coil and the matching loop Ls together with the passive T/R switch. The inductance of the coil is L_p_ ≈ 0.7 μH and the tuning capacitor is C = 2,080 pF (9 × 220 pF + 100 pF chip capacitors from ACT Corp.), L_s_ is the inductance of the matching loop.

For validation purposes, the first NMR experiments have been conducted at 0.1 T magnetic field created by a commercial electromagnet (Bouhnik S.A.S, Vélizy Villacoublay, France). The RF coil was a home-made transmit-receive 7-turns solenoid coil of 20 mm diameter and 16 mm length. The electrical circuit of this coil is shown in Figure 7, together with the passive T/R switch.

The inductance *L_p_* of the coil and the tuning capacitor *C* were about 0.7 μH and 2,080 pF, respectively. No adjustable variable capacitor section was used for fine tuning the coil at this validation stage. Actually, the power supply of the used magnet allows to slightly change the value the magnetic field around 0.1 T. The working frequency of the DDS and the SDR, which is fully programmable, is then easily adjusted to fit the resonance frequency of the coil (when this is inside the magnet and loaded by the sample). In other words, we perform a tuning of the working frequency rather than a tuning of the resonance frequency of the coil. On another hand, this solution avoids the use of somewhat sized variable capacitors.

The coil was matched to 50 Ω using an inductive coupling between the inductance L_P_ of the coil and the inductance L_S_ of a second loop. In this design, the matching is performed for each sample by moving the loop with respect to the coil [17]. One of the advantages the inductive coupling is that the electrical balancing of the probe with respect to the electric field is automatically obtained. By doing so, the dielectric losses due to capacitive coupling between the sample and the coil are reduced [18]. These losses could not be neglected even at 4.2 MHz, especially with samples that could have a poor dielectric quality. Moreover, by using an inductive coupling, the tuning of the coil is almost unaffected by various loads (samples). Only the matching is changed. More generally, the tuning and the matching could actually be realized independently.

Note that the electrical balancing of the coil could be also obtained by symmetrical capacitive coupling networks. However, at 4.2 MHz the use of the inductive coupling avoids the use large values and sized capacitors (especially adjustable ones) which could be not compatible with the available space inside the magnet.

The passive switch was based on a conventional quarter-wave λ/4 transmission line approach. The use of this approach is acceptable in this validation phase. However, due to the long length of the transmission line at such relatively low working frequencies (λ/4 ≈ 12 m at 4.2 MHz), it could be more appropriate to replace this line by a lumped-elements quarter-wave phase shifter in a final system design.

**Figure 8.**
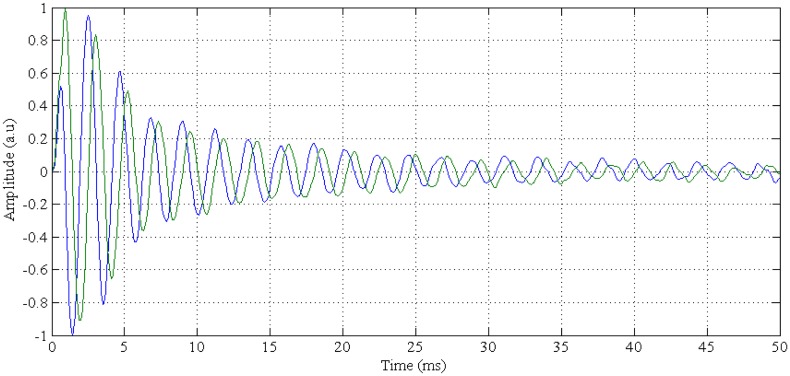
Base band I/Q signals of the free induction decay (FID) obtained with a hard 90° pulse of 100 μs of duration and 50 ms of repetition time.

First NMR signals were obtained under these conditions. The sample was a tube of about 1 cm^3^ of water. Figure 8 shows the I/Q base band signals of the Free Induction Decay (FID) with a small amount of off-resonance (400 Hz). The excitation was a hard 90° pulse with a width of 100 μs. The repetition time, TR, was about 500 ms.

While these first results show a proof-of-concept of the use of SDF and DDS in dedicated home-built low-field NMR systems, it is however necessary to proceed well beyond this demonstration of feasibility in a future work. This work should be focalized on several directions. Firstly, it is important to qualify the SNR of the whole NMR system (including the coil and the preamplifier). The idea is to explore the different possibilities for the optimization of the performances in NMR experiments. Upon the obtained noise performances, this optimization may concern the new designs for the digital filters and the choice of another VGA with better noise performances. Secondly, the impact of the AGC operation must also be evaluated. We expect that this automatic gain control should at least contribute in the enhancement of the SNR of the NMR signals.

Other direction in our work should concern the implementation of the NMR/MRI sequences for various applications. In addition to classical spin- and gradient- echo sequences (which should also confirm the proof-of concept), the Carr-Purcell-Meiboom-Gill (CPMG) sequence will also be investigated for the application of moisture measurement of nuts. More specialized sequences for the high resolution NMR in inhomogeneous field are should be implemented in a near future. The spectrometer is easily transportable and should be suitable for use in mobile and single-sided NMR instruments.

## 5. Conclusions

We have presented a new electronics and associated software for low-field NMR systems. In the proposed design, we replaced as many analog components as possible with digital electronics and software. Actually, except for the RF power amplifier and the low-noise preamplifier, the whole system is digital, including a DDS for pulse generation, a SDR pour digital signal receiving and a DSP for pulse programming. The first results have validated the use of these telecommunication concepts for the NMR. The flexibility of the system should allow its use, without hardware and software modification, for large palette of NMR applications, especially for mobile and *in-situ* NMR at low-field.
